# A Probable Case of Tirzepatide-Induced Acute Pancreatitis

**DOI:** 10.7759/cureus.85291

**Published:** 2025-06-03

**Authors:** Yovan Sumaruth, Rudramun Gopal, Faris Chater, Saurav Dhawan, Tamzin Wild

**Affiliations:** 1 Internal Medicine, North Manchester General Hospital, Manchester, GBR; 2 Internal Medicine, Salford Royal Hospital, Salford, GBR

**Keywords:** acute pancreatits, glp-1 receptor agonist, medication-induced pancreatitis, multiple gallstones, tirzepatide

## Abstract

Glucagon-like peptide-1 (GLP-1) receptor agonists have become increasingly popular for their dual benefits in glycaemic control and weight reduction. However, emerging concerns have been raised regarding their association with acute pancreatitis. Tirzepatide, a novel dual GLP-1/gastric inhibitory polypeptide (GIP) receptor agonist, is currently a subject of concern. We report the case of a 32-year-old woman with a prior history of gestational diabetes mellitus, who presented with a three-week history of worsening epigastric pain, nausea, vomiting, and constipation. The patient had commenced tirzepatide (Mounjaro) five weeks earlier for weight loss, with five doses administered prior to admission. She had received 2.5 mg subcutaneously weekly for four weeks, followed by one dose of 5 mg subcutaneously. Examination revealed severe epigastric tenderness without peritonism. Laboratory tests showed markedly elevated lipase (11,645 U/L), transaminases, alkaline phosphatase, and bilirubin levels. Imaging findings were consistent with acute interstitial oedematous pancreatitis and revealed incidental gallstones without signs of choledocholithiasis or cholecystitis. CT and magnetic resonance cholangio-pancreatography (MRCP) confirmed pancreatic inflammation without biliary obstruction. The patient was managed conservatively with cessation of tirzepatide, supportive care, and close monitoring. She showed significant clinical improvement with normalisation of lipase levels by discharge. This case raises concerns over the potential risk of pancreatitis associated with tirzepatide use. Although the patient's pancreatitis may appear to be most consistent with gallstone-induced aetiology, the recent initiation of tirzepatide may be a potential contributing factor, as the strong temporal correlation between drug initiation and symptom onset, coupled with clinical resolution upon discontinuation, suggests a probable causal relationship. Clinicians should maintain a high index of suspicion for drug-induced pancreatitis in patients using GLP-1 receptor agonists, including tirzepatide, especially in those with pre-existing metabolic risk factors or gallstones. This case underscores the need for continued surveillance and more robust data to guide the safe use of tirzepatide in routine clinical practice.

## Introduction

Tirzepatide is the first dual co-agonist for glucose-dependent insulinotropic polypeptide (GIP) and glucagon-like-peptide-1 (GLP-1) receptors used in the treatment of type 2 diabetes and weight loss [1]. It is given as a once-weekly subcutaneous injection and results in weight loss and improved glucose control via stimulating release of insulin from the pancreas, and leads to increased adiponectin levels, thereby reducing hyperglycaemia [2]. Results from a post-hoc analysis encompassing the composite end points across the five clinical trials, SURPASS 1-5, show a significant improvement in glycaemic control and weight loss without hypoglycaemia compared to placebo, semaglutide, insulin degludec and glargine [3]. 

## Case presentation

A 32-year-old female presented to the Accident & Emergency department with a three-week history of abdominal pain that had worsened in severity one day prior to admission. The pain was described as severe, sharp in character, and more pronounced around the epigastric region. It was also associated with nausea, vomiting, and constipation. She had a history of gestational diabetes mellitus and obesity (BMI 40.5 kg/m^2^) for which she had started taking tirzepatide (Mounjaro) for weight loss five weeks previously. She had commenced 2.5 mg subcutaneous once weekly for four weeks, followed by a dose of 5 mg subcutaneous in the week leading to admission. Regarding her social history, she denied use of alcohol, had no smoking history, or use of recreational drugs. 

On examination, her abdomen was soft with severe epigastric tenderness but no evidence of guarding or rigidity present. Investigations revealed an elevated serum lipase of 11,645 U/L (reference: 13-60 U/L), alanine transaminase 514 IU/L (reference: 1- 35 IU/L), alkaline phosphatase 758 U/L (reference: 30-130 U/L), bilirubin 41 umol/L (reference 0-21 umol/L), and adjusted calcium 2.67 mmol/L (reference: 2.20-2.60 mmol/L) (Table 1).

**Table 1:** Laboratory results at admission

Investigation	Patient Value	Reference range
Lipase	11,645 U/L	13-60 U/L
Alanine transaminase	514 IU/L	1-35 IU/L
Alkaline phosphatase	758 U/L	30-130 U/L
Bilirubin	41 umol/L	0-21 umol/L
Adjusted Calcium	2.67 mmol/L	2.2-2.6 mmol/L

**Figure 1:**
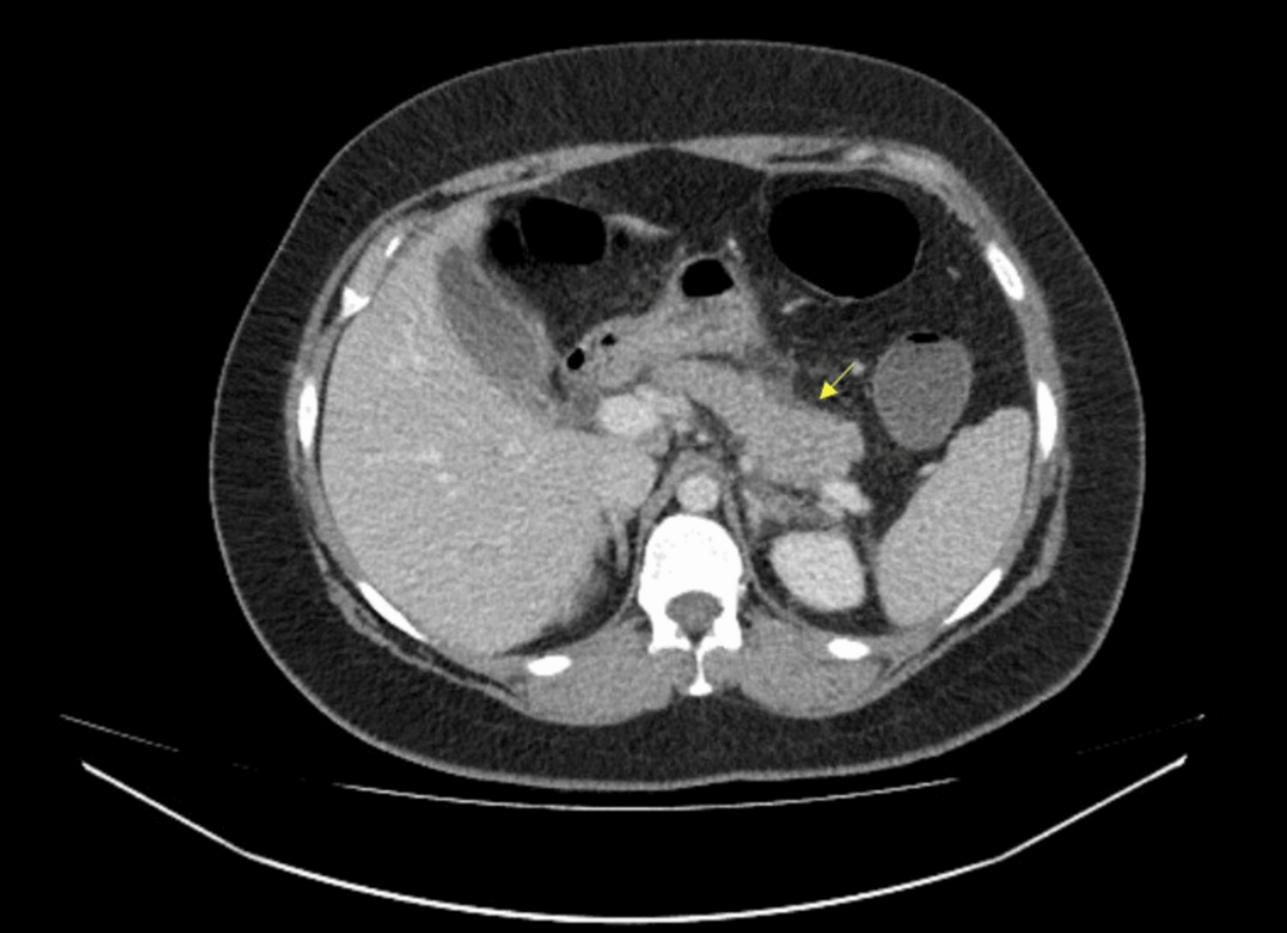
CT Abdomen and Pelvis with contrast The distal head and tail are bulky with peripancreatic inflammatory changes and peripancreatic fluid. Imaging features are in keeping with mild acute interstitial oedematous pancreatitis and mild ascites.

Ultrasound was performed, which showed the gallbladder was thick-walled and contained a small amount of echogenic debris and several small calculi at the neck. Overall appearances were consistent with acute cholecystitis. The pancreas was obscured by bowel gas and could not be assessed. A computed tomography (CT) scan of the abdomen and pelvis subsequently revealed that the distal head and tail of the pancreas were bulky with peripancreatic inflammatory changes and peripancreatic fluid, tracking to the left anteroconal fascia, mild fluid in the pelvis, and tiny uncomplicated gallstones. The imaging features were in keeping with mild acute interstitial oedematous pancreatitis and mild ascites (Figure 1).

The patient was reviewed by the surgical team, who advised a magnetic resonance cholangiopancreatography (MRCP) scan, which revealed imaging features in keeping with acute interstitial oedematous pancreatitis with multiple tiny uncomplicated gallstones seen but no evidence of cholecystitis. There were no other significant abnormalities identified. 

During her hospital admission, there was a rise in the C-reactive protein (CRP) from 22 mg/L to 278 mg/L (reference: 0-5 mg/L) with a procalcitonin level of 0.05 ug/L (reference <0.5 ug/L), which suggested an inflammatory response rather than an infective picture. Additionally, her lipase levels dropped from 11645 U/L to 66 U/L (reference: 13-60 U/L) over her stay. The Naranjo Adverse Drug Reaction Probability Scale [4] was calculated, and the patient scored 5 out of 10.

The patient was treated for acute interstitial pancreatitis, probably secondary to the use of tirzepatide in the context of tiny, uncomplicated gallstones without features of cholecystitis on MRCP. She required multiple analgesic options, including low-dose paracetamol, codeine, and oral morphine to manage her pain in addition to intravenous fluids and encouragement of oral nutrition over the course of her inpatient stay. Her admission to the hospital resulted in her missing her sixth tirzepatide weekly due dose, and following this, her pain began to gradually improve. Upon discharge, she was advised to stop using tirzepatide and made a good clinical recovery.

## Discussion

This case brings to light a critical concern in contemporary clinical practice: the potential risk of acute pancreatitis associated with the use of GLP-1 receptor agonists, particularly tirzepatide. The patient, a 32-year-old woman with a history of gestational diabetes mellitus, developed acute pancreatitis soon after commencing tirzepatide for weight management. The close temporal relationship between the initiation of the medication and the onset of symptoms, along with the marked improvement following the discontinuation of the drug, strongly implicates tirzepatide as a likely causative agent. Additionally, on the Naranjo scale [4], she scored 5 out of 10, indicating the probable likelihood of a drug-induced adverse reaction.

Nevertheless, the safety profile of tirzepatide, particularly in relation to pancreatitis and gallbladder or biliary disease, remains insufficiently explored. Zeng et al., when conducting a systematic review and meta-analyses of nine randomised control trials, found no significantly increased risk of pancreatitis for patients receiving tirzepatide compared with control groups (basal insulin, GLP 1 receptor agonists, or placebo) [5]. Moreover, in a large phase 3 double-blind randomised control trial by Jastreboff et al. assessing the safety profile of tirzepatide, their findings reported only three cases of pancreatitis, one each in the 5 mg, 10 mg, and 15 mg tirzepatide groups, underscoring both the rarity of this adverse event and the need for vigilance [6]. However, whilst tirzepatide may generally be considered safe in terms of pancreatitis, a subgroup analysis by Zeng et al. revealed a statistically significant association between the use of 10 mg tirzepatide and subsequent increased risk of gallbladder or biliary disease [5].

Overall, the current paucity of robust and comprehensive data highlights the pressing need for further research into the long-term safety of tirzepatide, especially in patients with type 2 diabetes mellitus and obesity. Such research is essential to better inform clinical decision-making and mitigate potential risks associated with this promising yet complex therapeutic agent.

GLP-1 Receptor Agonists and the Risk of Pancreatitis

GLP-1 receptor agonists have revolutionised the management of type 2 diabetes mellitus, offering the dual benefits of improved glycaemic control and weight reduction in a dose-dependent manner [7-9]. The association between GLP-1 receptor agonists and pancreatitis first garnered attention following post-marketing surveillance reports and subsequent observational studies. The European Medicines Agency product information for Mounjaro (tirzepatide) includes a section on special warnings and precautions, mentioning that acute pancreatitis has been reported in patients treated with tirzepatide, and cautioning its use in patients with a history of pancreatitis [10]. A notable study by Singh et al. in 2013 showed an increased odds of hospitalisation with acute pancreatitis among users of GLP-1-based therapies [11]. However, a meta-analysis conducted by Monami et al. found no significant increase in the risk of acute pancreatitis among users of GLP-1 receptor agonists compared to other antidiabetic therapies [12]. Additionally, in a systematic review and meta-analysis of real-world data by Wang et al. that analysed seven retrospective cohort studies, no association was found between incretin-based therapies and risk of acute pancreatitis [13]. However, these results should be interpreted with caution, given the small number of studies analysed and the confounding nature of observational data. Furthermore, although the absolute risk remains low, the findings have prompted calls for greater vigilance and more extensive research into the safety profile of these drugs. This approach extends to adequately counselling patients on the potential side effects, including gastrointestinal effects and epigastric pain [14].

In this case report, the presence of gallstones complicates the clinical picture, as gallstone disease is a well-established cause of acute pancreatitis. However, the absence of choledocholithiasis and the rapid improvement in the patient’s condition following the cessation of tirzepatide strongly suggest that the drug was the primary trigger for pancreatitis. This observation aligns with findings from Drucker et al., who noted that GLP-1 receptor agonist-associated pancreatitis often occurs in the absence of classical risk factors, suggesting that the drug itself may induce pancreatic inflammation independent of other etiologies [15]. 

Diagnostic and Therapeutic Considerations

The diagnosis of drug-induced pancreatitis is inherently challenging, particularly in patients with multiple potential risk factors, as was the case here. The patient presented with severe epigastric pain, markedly elevated lipase levels, and imaging findings consistent with acute interstitial pancreatitis. While the initial ultrasound findings raised the possibility of acute cholecystitis, subsequent imaging with CT and MRCP provided a clearer picture, confirming the diagnosis of pancreatitis without evidence of biliary obstruction. This highlights the critical role of advanced imaging modalities in accurately diagnosing pancreatitis and differentiating it from other abdominal pathologies [16]. The utility of MRCP in this context is well-documented, with reviews such as those by Griffith et al. emphasising its value in assessing pancreatic and biliary structures, particularly when ultrasound findings are inconclusive [17].

From a therapeutic standpoint, the management of drug-induced pancreatitis necessitates the prompt discontinuation of the offending agent. The patient’s presenting symptoms after two doses of tirzepatide and the swift clinical recovery following the cessation of tirzepatide, not only reinforce the critical importance of a detailed medication history in patients presenting with acute pancreatitis, but also highlight the necessity for early identification of potentially causative medications. The National Institute for Health and Care Excellence (NICE) acknowledges that acute pancreatitis has been reported in people having tirzepatide treatment and recommends immediate stopping of tirzepatide if symptoms of acute pancreatitis are seen, and it should not be started if pancreatitis is confirmed [18]. This case, therefore, emphasises the urgent need for more precise and evidence-based protocols to guide clinicians in mitigating the risks associated with GLP-1 receptor agonists, ensuring safer patient outcomes.

## Conclusions

Although the risk of drug-induced pancreatitis appears to be low, it may be underreported due to the often mild and self-limiting nature of the condition and the presence of atypical symptoms. Therefore, clinicians should maintain a high index of suspicion for drug-induced pancreatitis, particularly when assessing patients using GLP-1 receptor agonists. Careful medication reconciliation and a detailed medical history are essential to identify risk factors that may precipitate or exacerbate pancreatitis.

Establishing a direct causal relationship between GLP-1/GIP receptor agonists and pancreatitis can be challenging, especially in patients with confounding factors such as incidental gallstones or a history of mild alcohol consumption. Nevertheless, it remains crucial to report suspected cases. Reporting is important because these medications may directly contribute to or worsen pancreatitis, thereby posing increased risks to patient safety. Furthermore, systematic reporting enhances drug safety surveillance, strengthens pharmacovigilance, and informs regulatory decisions
